# Have we made progress in Somalia after 30 years of interventions? Attitudes toward female circumcision among people in the Hargeisa district

**DOI:** 10.1186/1756-0500-6-122

**Published:** 2013-03-27

**Authors:** Abdi A Gele, Bente P Bø, Johanne Sundby

**Affiliations:** 1 Department of Social Science, Oslo and Akershus University College of Applied Science, Pilestredet 35, Oslo 0167, Norway; 2 Department of General Practice and Community Medicine, Institute of Health and Society, University of Oslo, Kirkeveien 166, Oslo, 0450, Norway

## Abstract

**Background:**

Female circumcision is a major public health problem that largely contributes to the ill-health of women and their children globally. Accordingly, the international community is committed to take all possible measures to abolish the practice that is internationally considered to be absolutely intolerable. While the practice is a social tradition shared by people in 28 African countries, there is no country on earth where FC is more prevalent than in Somalia. Yet, since the early 1990s, there is no quantitative study that has investigated whether the perception towards the practice among Somali men and women in Somalia has improved or not. Thus, this cross-sectional quantitative study examines the attitudes toward the practice among people in Hargeisa, Somalia.

**Methods:**

A cross-sectional study of 215 randomly selected persons, including both men and women, was conducted in Hargeisa, Somalia from July to September of 2011. Participants were interviewed using structured questionnaires, with questions including the circumcision status of the female participants, the type of circumcision, if one has the intention to circumcise his/her daughter, whether one supports the continuation or discontinuation of the practice and men’s perceptions toward having an uncircumcised woman as a wife.

**Result:**

The findings show that 97% of the study’s participants were circumcised with no age differences. Of this, 81% were subjected to Type 3, while 16% were subjected to either Type 1 or 2 and only 3% were left uncircumcised. Approximately 85% of the respondents had intention to circumcise their daughters, with 13% were planning the most radical form. Among men, 96% preferred to marry circumcised women, whereas overall, 90% of respondents supported the continuation of the practice. The vast majority of the study’s respondents had a good knowledge of the negative health effects of female circumcision. In multivariate logistic regressions, with an adjustment for all other important variables, female circumcision (the Sunna form) is a religious requirement 16.5 (2.43-112.6) and the *Sunna* form is not harmful 25.1(2.35-281.1), are the two factors significantly associated with the continuation of female circumcision. Moreover, females were less likely to support the continuation of FC compared to their male counterparts (aOR 0.07; CI: 0.05-0.88).

**Conclusion:**

The study shows that the support towards the persistence of the practice is profoundly high in Somalia. People are aware of the health and human rights effect of female circumcision, and yet they support the continuation of the practice. Therefore, over 30 years of campaigns with limited progress demand an alternative approach towards the eradication of female circumcision in Somalia.

## Background

Over the past four decades, the traditional removal of vital and normal external genital tissue of girls (called female circumcision (FC) and female genital mutilation or cutting (FGM\C) has become a major concern, and there has been an international consensus to take all possible measures to abolish a practice that is internationally deemed as a serious human rights and public health problem that concerns all sectors of society [1]. In the face of this international consensus about the protection of the health and human rights of children and women, with few exceptions, almost all the girls born in Somalia today undergo FC [2,3]. The key to FC abandonment efforts is an understanding of the attitude towards the practice among practicing communities. Yet, since the early 1990s, with the exception of the multiple indicator cluster survey (MICS), which only targeted women between the age of 15–49 [3], there has been no quantitative study that has investigated whether or not the perception towards the practice among Somali men and women in Somalia has improved in relation towards its abandonment.

Female circumcision is a traditional surgery that is usually performed on girls between the ages of 4–10 by a layperson with limited anatomical knowledge and medical training [4]. Practitioners perform the surgery often, which usually involves one of four types classified by the WHO [5]. Type I, also called *clitoridectomy*, involves the partial or total removal of the clitoris, while Type II (*excision*) involves the partial or total removal of the clitoris and labia minora. Type III, which is called *infibulation* is the most radical form, and it involves a partial or total removal of the external genitalia and a sealing of the vaginal opening, leaving only a small hole for urine and menstrual blood to pass. Type IV involves all of the other harmful procedures to the female genitalia for non-medical purposes, such as pricking, piercing, etc. Female circumcision is most prevalent in 28 countries in Africa, but is also found in Asia and Western countries that host immigrants from areas with traditions of FC [6]. An estimated 100 to 140 million girls and women have undergone the practice, with approximately three million girls being subjected to the practice every year [7]. Due to its severe negative health impact, FC is internationally considered as a ritualized form of child abuse and violence against women [8]. The gravity of the health complications of FC vary according to type, but even in its mildest form, it constitutes an unacceptable health risk/damage and violation of human rights [9].

An early study indicated that 85% of circumcised women are likely to be affected by a condition that requires medical attention at some point in their lifetime [10]. Some immediate health complications that girls may experience during the procedure are extreme pain, excessive bleeding, shock, septicemia, tetanus and infections [7,11], with other obvious complications including the retention of urine, difficulties in menstruation and impaired sexual pleasure [12]. Furthermore, invasive forms of FC can also cause increased risks of complications in childbirth, including cesarean sections, episiotomy, perineal tears, postpartum hemorrhage and an increased risk of stillbirths and neonatal deaths [13,14]. Additionally, increased risks of cervical cancer [15] and death have also been associated with this practice [16].

In addition to the negative health consequences of FC, it is vital to highlight that the practice reflects a gender inequality that establishes an extreme form of female discrimination. Progress towards its abandonment may therefore contribute to the empowerment of women (MDG 3), an improvement of maternal health (MDG 5) and a reduction in child mortality (MDG 4). Accordingly, for the good health and human rights of women and children, the United Nations has denounced all forms of the practice, rejecting any shift towards accepting milder forms as well as towards the medicalization of the practice [17].

### Female circumcision in Somalia

Being Somali entails all of the Somali-speaking people who inhabit the Horn of Africa, including Somalia, the northeastern region of Kenya, the Somali region of Ethiopia and the Republic of Djibouti. Somalis are the most homogeneous group of people in Africa, with 98% of Somalis speaking the Somali language and sharing a similar culture and traditions [6].

Female circumcision has been practiced by the Somali people since time immemorial, and while the practice is a tradition shared with people in 28 African countries, there is nowhere on earth where FC is more prevalent than in Somalia [2]. The Somalis classify the practice into two forms: the so-called *Sunna* form (*gudniinka sunniga ah),* which comprises anything that is less than infibulations, and the most drastic form, namely *Pharoanic* (*gudniinga fircooniga ah),* which comprises Type III [2,18]. This practice is often performed on Somali girls between the ages of 4–10 by a medical practitioner, midwife, or most often by a traditional practitioner from a family in which generations of that family have been traditional practitioners. Unlike other parts of the world where the milder forms of FC (Type I and II) are predominant and comprise 85% of all FC, the vast majority of girls in Somalia are infibulated (80-90%) [2,3]. Female circumcision has been known to be ethnically affiliated, yet in Somalia it is practiced by all clans. However, there is a slight variability among clans in terms of the type of FC they practice. Infibulation is more prevalent among pastoralist-dominated clans in the northern and central parts of the country than in the farming communities of the south [3]. According to Gallo *et al.*, infibulations were almost absent among southern farming clans in the early 19th century, while it was over 95% in northern pastoralist clans [19].

Beginning in the 1970s, the former Somali regime openly took a stance against FC, and backed numerous campaigns aimed at eliminating the practice using a variety of approaches. The Somali Women’s Democratic Association (SOWDA) was founded to implement anti-FC projects designed to eradicate the practice in Somalia. Even so, all government efforts against FC collapsed with the overthrow of the military regime in 1991, and since then, the FC programmes in Somalia have relied on efforts by international and local women’s organizations. These programmes have usually been implemented with little effort towards evaluating their impact on the community’s attitude and behaviour toward the practice [2]. The reason for this is mainly because many of the implementing organizations may not be familiar with the essential research methods and principles of experimentation necessary for evaluating whether or not the intervention programmes work, in addition to how and why they work [20]. As a result, there has been a widely expressed doubt about whether decades of programming efforts aimed at eliminating FC in Somalia have had much success in enabling an attitude change towards this practice [18]. Accordingly, the only available literature based on research conducted in the 1980s and 1990s reveals a universal prevalence of FC in Somalia, with 85% of urban and 98% of rural women undergoing the severest form [21-23]. Moreover, a report shows that 90% of Somali women support the continuation of this practice [2].

A prior study indicates that contemporary social change, including an improved education, urbanization and increased awareness about the harmful effects of the practice, affects the perceptions of people towards the practice [24]. Nonetheless, over three decades have passed since the effort to end FC began in Somalia, and the question of whether or not Somalis’ attitude towards FC has improved is as relevant today as it was in the 1980s. Therefore, as part of a broad-based study on FC among Somalis in Norway and in Somalia, this quantitative study investigates whether or not the traditional positive attitudes toward FC among Somali men and women in the Hargeisa district have improved.

## Methods

A cross-sectional study using a systematic random sampling was carried out in Hargeisa from July to September of 2011. One of the six counties in the town, namely Mohamud Haybe, was selected using random numbers. Afterwards, from an estimated total of 1,000 households in the county, every 4th house was selected. One person was interviewed from each household until the desired sample size of 215 was achieved, with the participants included in the study needing to be ≥18 years, permanently living in the household and verbally consenting to be interviewed. We followed common research ethics principles in the carrying out of this study, including informed consent, the right to refuse, as well as withdrawal and confidentiality. This study was ethically cleared by both the Norwegian Ethical Committee and the Ethical Committee of the Ministry of Health of Somaliland.

A pretested structured interview-questionnaire was used for this study, with open-ended questions also being asked that were later quantitatively categorized. The questionnaires were developed into English, translated into Somalia and then back-translated into English before developing the final version in Somali, which is the native language of the researcher, assistants and respondents. The questionnaires were administered by two female and two male university graduates, all of whom received training on the data collection tools by the researcher.

The questionnaires included socio-demographic details, as well as questions that assessed people’s knowledge and attitude towards FC. The dependent variable was whether one supports the continuation or discontinuation of one form and/or all forms of FC, with the knowledge questions including questions that address the health effects of FC, i.e. whether FC leads to bleeding, infection, complications in child birth, difficulties in urination and the possible transmission of HIV. Questions that assess the attitude towards FC include: Is FC a religious requirement? Is it a harmful culture? Does it protect the honor of girls? Does it prevent adultery? Does it lead to trustable marriage, etc.? All of these questions were categorized into four categories: “Yes for the Sunna form, yes for the Pharaonic form, yes for both forms and yes for none of the forms”. Moreover, female participants were asked if they themselves had been circumcised and the type of their circumcision, which was categorized as S*unna* or *Pharaonic*. By contrast, male participants were asked if they would prefer to marry women who were circumcised or uncircumcised, and both males and females were asked if they circumcised their daughters or had intentions to do so and the reasons behind their decision.

### Analysis

The data was analysed using SPSS version 18, with Chi-squares used for analysing categorical variables and t-tests for the analysis of continuous variables. During the analysis, the four categories of questions on attitudes towards FC were collapsed into two categories; i.e. those who responded: yes, the *Pharoanic* form is a religious requirement; yes, the S*unna* form is a religious requirement and both forms are religious requirements were collapsed together and coded as “1”, while those who said that none of the forms were religious requirements were coded as “0”. All other questions that addressed the attitude towards FC were coded in similar way. The model fit to the data was determined through a Hosmer-Lemeshow goodness of fit test, whereas the proportional differences between men and women with respect to knowledge on the health effects of FC and the attitude towards the practice were determined using a Fisher’s exact test. A p≤0.05 was considered as statistically significant. Although some of the cells had observations as low as 2, yet we decided to run regression analysis to see the variables associated with attitude change towards the practice. Thus, a univariate logistic regression analysis was run. Afterwards, variables that were shown to be significant in univariate analyses were added into the multivariate analyses. The degree of association was measured by an odds ratio with a 95% confidence interval (CI).

## Results

A total of 215 persons were interviewed from July to September of 2011. The mean age of the study sample was 31 years, with no difference in the mean age between males and females. As shown in Table 1, the number of males and females in this study were almost equal, with a ratio of 1:1. Roughly one-third of the study participants had no formal education (33%), with approximately 20% having a primary education, 24% having a secondary education and 22% having a college or university education.

**Table 1:** Characteristics of the study participants (n=215)

**Variables**	**N**	**%**
**Gender**		
**Male**	108	50.2
**Female**	107	49.8
**Age**		
**≤25**	93	43.3
**26-40**	88	40.9
**>40**	32	14.9
**Education**		
**University/college**	47	21.9
**Secondary**	52	24.5
**Primary**	42	19.5
**No formal education**	71	33
**Marital status**		
**Single**	100	46.5
**Married**	84	39.1
**Divorced**	16	7.4
**Widowed**	15	7.0

Of the 107 female participants, 104 (97%) reported that they were circumcised, while only three (3%) reported that they were not circumcised (Table 2).

**Table 2:** Types of circumcisions of women participants

**Type of FC**	**N**	**%**
**Type III (Pharoanic)**	87	81.3
**Types I & II (Sunna)**	17	15.9
**Not circumcised**	3	2.8

When the participants were asked if they experienced any problems from their circumcision, 64 (60%) said yes, while 39 (36%) said they did not experience any such problems. Approximately one-quarter (28%) of female participants believed that being circumcised was/will be an advantage for the success of their marriage.

Out of the 212 persons who responded to the question of whether they intended to circumcise their daughters, 182 (85%) had the intention to perform this practice. Of these, 154 (71.6%) were planning to use the *Sunna* form, with the reason behind their decision either being because they considered the S*unna* form to be harmless or because it was seen as a religious requirement. The 28 (13%) participants who were planning to use the *Pharoanic* form believe that it is part of their culture, that it maintains the honour of the girls and that it prevents adultery. Of the 30 (14%) persons who reported that they will not circumcise their daughters, 22 (73%) were women and only 8 (27%) were men, with the difference being statistically significant (p<0.001).

Out of 108 men in this study, 104 (96%) preferred to marry circumcised women over uncircumcised ones. However, 92 (85%) preferred the Sunna form, 12 (11%) preferred the Pharoanic form and only 2.8% would choose uncircumcised women to be their wives.

The majority of study participants knew that FC caused bleeding (84.7%), difficulties in urination (81.4%), complications in child birth (73%), infection (76.7%) and the possibility of HIV transmission (88.8%). As shown in Table 3, with the exception of one variable, there was no significant gender difference in knowledge about the health effects of FC.

**Table 3:** Gender differences in the knowledge about the health effects of, and the attitude towards, the practice

**Variables**	**Men(%)**	**Women(%)**	**Total(%)**	**P-value**
**Knowledge variables**				
**Cause bleeding**				
**Yes**	95(90.5)	87(82.9)	182(86.7)	P<0.14
**No**	10(9.5)	18(17.1)	28(13.3)	
**Total**	**105**	**105**	**210**	
**Difficulty in urination**				
**Yes**	88(89.8)	87(87.9)	175(88.8)	P<0.41
**No**	10(10.2)	12(12.1)	22(11.2)	
**Total**	**98**	**99**	**197**	
**Difficulty in child birth**				
**Yes**	89(91.8)	68(80.0)	157(86.3)	P<0.02
**No**	8(8.2)	17(20.0)	25(13.7)	
**Total**	**97**	**85**	**182**	
**Infection**				
**Yes**	90(90.9)	75(90.4)	165(90.7)	P<0.53
**No**	9(9.1)	8(9.6)	17(9.3)	
**Total**	**99**	**83**	**182**	
**HIV transmission**				
**Yes**	103(97.2)	88(96.7)	191(97)	P<0.57
**No**	3(2.8)	3(3.3)	6(3)	
**Total**	**106**	**91**	**197**	
**Attitude variables**				
**FC prevent pre/extra marital sex**				
**No**	52(48.6)	87(82.1)	139(65.3)	P<0.001
**Yes**	55(51.4)	19(17.9)	74(34.7)	
**Total**	107	106	213	
**Is a harmful culture**				
**Yes**	1(0.9)	13(12.1)	14(6.5)	P<0.002
**No**	106(99.1)	94(87.9)	200(93.5)	
**Total**	107	107	214	
**Leads to a trustable marriage**				
**No**	54(51.9)	60(56.1)	114(54.0)	P<0.583
**Yes**	50(48.1)	47(43.9)	97(46.0)	
**Total**	104	107		
**Keeps the dignity of girls**				
**No**	52(49.1)	76(73.8)	128(61.2)	P<0.001
**Yes**	54(50.9)	27(26.2)	81(38.8)	
**Total**	**106**	**103**	**209**	
**Is religious requirement**				
**No**	4(3.8)	16(15.0)	20(9.4)	P<0.005
**Yes**	102(96.2)	91(85.0)	193(90.6)	
**Total**	**106**	**107**	**213**	
**Support the discontinuation of the practice**				
**Yes**	2(1.9)	21(19.6)	23 (10.7)	P<0.001
**No**	105(98.1)	86(80.4)	191(89.3)	
**Total**	**107**	**107**	**214**	

Approximately 90% (191) of the study participants supported the continuation of one or all forms of FC. Of this, 164 (76.3%) persons supported the continuation of the *Sunna* form only, while 10.7% supported the continuation of all forms and a similar proportion (23, 10.7%) supported the discontinuation of all forms of FC. As shown in Table 3, of the 23 participants who supported the discontinuation of all forms of FC, 21 (91.3%) were females, whereas only two (8.7%) were males, with this difference being statistically significant (p<0.001).

As shown in Table 4, education and age variables were not significantly associated with the continuation of the practice, although females were found to be less likely to support the continuation compared to males (OR=0.07(0.05-0.88). Similarly, the attitude factors associated with the continuation of FC were that the practice maintains the dignity of girls (OR=7.2 CI:1.661-32.205), that it is a religious requirement (OR=53.7 CI:16.329-176.37) and that FC is not harmful (OR=103.1 CI:20.5-518.7). However, that FC is required for engaging in a trustworthy marriage was not significantly associated with a continuation of FC (OR=1.7 CI:0.68-4.16). With an adjustment of other important variables, being a female was less likely to support a continuation of FC (aOR 0.07 CI: 0.05-0.88). Moreover, FC being a religious requirement (aOR=16.5 CI:2.43-112.6) and that FC is not harmful (aOR 25.1 CI :2.35-281.1) continue to be significantly associated with the continuation of FC.

**Table 4:** The factors associated with a continuation of the practice in Somaliland

**Support the continuation or discontinuation of FC?**				
**Variables**	Support the discontinuation of all forms of FC	Support the continuation of one or all forms of FC	Model 1	Model 2
			Unadjusted	Adjusted
**Gender**				
**Male**	2(1.9)	105(98.1)	1.00	1.00
**Female**	21(19.6)	86(80.4)	0.08(0.05-0.88)*)*	0.07(0.05-0.88)*
**Age**				
**≤25**	13(14)	80(86)	1.00	1.00
**26-40**	5(6)	83(94)	2.7(0.92-7.91)	3.37(0.64-17.6)
**≥41**	4(13)	27(87)	1.1(0.33-3.65)	4.52(0.30-69.1)
**Is a religious requirement?**				
**No**	14(70)	6(30)	1.00	1.00
**Yes**	8(4)	184(96)	53.6(16.32-176.4)*	16.5(2.43-112.6)*
**Keeps the dignity of girls?**				
**No**	20(15.6)	108(84.4)	1.00	1.00
**Yes**	2(2.5)	79(97.5)	7.31(1.66-32.20)*	3.05(0.37-33.08)
**Leads to a trustable marriage?**				
**No**	15(13.2)	99(86.8)	1.00	
**Yes**	8(8.2)	89(91.8)	1.68(0.68-4.16)	
**The FC 1s a harmful culture?**				
**Yes**	12(86)	2(14)	1.00	1.00
**No**	11(5.5)	189(94.5)	103.1(20.49-518.7)*	25.1(2.35-281.1)*

Model 1: Unadjusted.

Model 2: All variables that were significant in Model 1, as well as the age variables, were entered in Model 2.

## Discussions

After more than four decades of anti-FC campaigns in Somalia, this study was conducted to estimate the attitude towards FC among Somali men and women in Hargeisa. The findings of this study provide important background information on the opportunities for intervention and the formulation of efficient FC programmes in Somalia. The results show that 97% of female study participants may be circumcised, with the vast majority (81.3) being subjected to infibulations. This finding is very similar to the findings of studies conducted in Somalia in the mid-1980s and early 1990s, which showed that 99% and 100% of Somali women were circumcised, respectively, with the vast majority being subjected to infibulations [21,22]. Generally speaking, 25% of women subjected to infibulations may eventually suffer from serious health complications [25]. The fact that 97% of Somali women in Hargeisa are circumcised today is an indication of the discouraging course of FC programmes in Somalia. Any progress towards the abolishment of FC is internationally deemed as being crucial to the empowerment of women (MDG 3), the improvement of maternal health (MDG 5) and a reduction in child mortality (MDG 4). Therefore, a critical review in FC programmes in Somalia may be needed to help this country progress towards achieving the aforementioned millennium goals, while simultaneously improving the health and human rights of women and children.

The other finding worth highlighting is the fact that the vast majority of study participants (85%) intended to circumcise their daughters, with the majority planning to perform a *Sunna* circumcision. The reasons forwarded in circumcising girls are that *Sunna* circumcision is not harmful, in addition to its religious obligation. This study is consistent with our recent study in Hargeisa and Galka’ayo (yet to be published), which reported an attitude shift from the *Pharoanic-* to the *Sunna* form in Somalia. Although there has been a widespread perception of a shift to a milder form (*Sunna*) in Somalia, only 16% of women were found to be circumcised in this form, with no differences among different age groups. The age composition of the sample may reflect a practice that took place 10–20 years ago, so to estimate current practice, one would have to investigate only children and teenagers. Clearly, the continuation of FC in Somalia has more to do with a religious misperception that concerns a stronger belief that the *Sunna* form is a religious obligation than any other justification. Supporters of Sunna circumcision generally use a single hadith as a justification for their argument. The hadith says that the prophet met a woman who was a circumciser and he said to her, “*Do not cut too severely, as that is better for a woman and more desirable for a husband.”* Nevertheless, many religious scholars regarded this passage as having little credibility or authenticity. In any case, the Koran clearly rejects any alteration of the human body from the way God created it. Female circumcision is a controversial topic within Muslim circles, and the important point to note is that Islam safeguards women’s rights to sexual enjoyment and health, and if female circumcision violates those rights, it would automatically be considered forbidden. In Somalia, where people believe that FC is practiced by all Muslims, a religious approach that is based on educating people about the position of Islam in the practice may work well in helping to abolish the religion-related perception towards FC. The members of the Somali immigrant community in Norway who have abandoned the practice have justified their attitude change in relation to an increased awareness about the un-Islamic nature of the practice [26]. The experience primarily gained from Somalis in Norway demonstrates that FC abandonment among Somalis is possible in less than one generation, provided that a number of culturally sensitive measures are adopted [26].

The present study shows that FC entertains high levels of support in Somalia, with almost 90% of the people in Hargeisa supporting the continuation of one or all forms of FC, which is higher than the proportion reported by the old literature in Somalia that shows an 80% support for the continuation of FC among Somali women [22]. However, the percentage of people supporting the practice is comparable with other studies performed in Somalia [27,28]. This result presents discrepancies with the result by multiple indicator cluster survey (MICS), in which only 32% of women in the North-West region of Somalia, which is the same region where we conducted this study, supported the continuation of the practice [3]. This difference is clearly due to the different ways that questions were framed by the studies. In this study, the question addressing people’s support towards the continuation or discontinuation of FC was categorized by the type of FC, such as *Sunna* and *Pharaonic*, whereas the MICS asked about people’s support towards FC and categorized the answers as yes/no, without any consideration of the Somali’s understanding of the term FC only referring to the *Pharaonic* type and excluding the *Sunna* type. A qualitative study conducted in Hargeisa, which will be published elsewhere, showed that when people were asked about their support towards FC, the majority supported its discontinuation, but when separately asked about their support towards the *Sunna* form, almost all supported its continuation. Thus, if the question about the support towards FC is not categorized by type, at least in a Somali context, one may risk drawing the wrong conclusions.

The other difference between our study and the MICS may be because we included men in this study, while the MICS was limited to women between the ages of 15–49. The reason for including men in this study was because more Somali men in the UK [6] and Norway [29] were reported to support the continuation of the practice than women. The high support of this practice in Somalia is not unexpected due to the fact that, except for an apparent shift from the *Pharoanic* to *Sunna* form in recent years, the achieved progress towards the abandonment of the practice in Somalia has been known to be very limited [2]. While an attitude change towards the practice regarding the harmful effects of the *Pharoanic* form on health is true in Somalia, the shift to the *Sunna* form may not be taken as progress unless we fully understand about the real definition of the sunna form. A prior study postulated that defending the milder forms of FC, which is a common behaviour in some African countries, means a resistance to change by people who have not accepted the need for all forms to be eliminated [9]. The issue of concern is that this study only used verbal classifications of the various forms, but not the extent of the cut involved in each type. Previous studies in Sudan have demonstrated that even forms called “Sunna” may be more severe than the definitions allow [30]. Nonetheless, a qualitative study that will be published elsewhere shows that the most prevalent Sunna cut in Somalia involves the removal of clitoris, either partially or totally, followed by two stitches. When the operation involves stitching, the WHO classifies it as Type III [5] regardless of the number of stitches. In another finding in Somalia, most parents asked their infants to undergo the larger excisions [31], thus further supporting the qualitative study finding.

Among the factors strongly associated with the continuation of FC in this study are that the *Sunna* form is a religious requirement and is not harmful. Although few Somali religious scholars in Western countries publicly reject the practice and have advocated against it [32], many religious leaders in Somalia defend the milder forms of the practice, with claims that the *Sunna* form is a religious requirement and is harmless [9]. As noted by Shanti, the information received by Somalis from religious institutions regarding FC is that the “*Sunna* circumcision is good because it does not have health problems, it is allowed by religion and it should be continued” [33]. These myths have created a false sense of religious justification to a practice that is an infringement on the physical integrity of women and girls. Consequently, a widespread belief that the *Sunna* form is a religious obligation prevails in Somaliland, and that any support to total eradication may mean disobeying one’s religion [33].

The study shows that people have a very good knowledge on the adverse health effects of the practice. A study by the WHO showed that an infibulated 15-year-old girl will lose nearly one-fourth of a year of her life and impose a financial burden on the health-care system [34]. The health consequences of FC are high, but it may be disproportional to the adverse social and cultural consequences that, if left uncircumcised, girls and their families may endure. This is the reason that, despite a very good knowledge of the health consequences of FC, most participants either subjected their daughters to FC or they had intentions of doing so.

One of the main findings in this study is the fact that men are more likely to support the continuation of FC than females, and the vast majority of them (96%) intend to choose a circumcised woman as a wife. A prior study among Somali youth in the UK found that males are more likely to support the continuation of FC [6]. Even so, a recent qualitative study among Somalis in Oslo reports that Somali men in Norway tend to prefer marrying uncircumcised women [26]. The difference in relation to the attitudes toward FC between Somali men in Somalia and their counterparts in Norway may be partially explained by factors related to the social environment in Norway, which is different than the social context in Somalia. Still, men’s attitudes toward FC can be one of the major bottlenecks in FC programmes in Somalia, where the issue of FC is widely considered as a women’s issue. Unlike other African countries such as Kenya, Gambia, Mali and Senegal, where men are considered as major stakeholders in FC programmes, Somali men are rarely or never recruited in the participation of FC campaigns. In Somalia, almost all FC campaigns are run by women’s organizations, whose main target audiences are often other women [2]. A prior qualitative study in Mali and Burkina Faso indicates that men recognize that this practice will not be abandoned without their involvement [35]. In Somalia, fathers play a critical role in determining all decisions concerning their family. At the grass-roots level, chiefs and clan elders, who are often men, decide the matters that concern their community. At the political level, it is community politicians, also often men, who decide the community issues. For Somali immigrants in Norway, where a considerable abandonment of FC was reported [26], Somali men played a crucial role in the achievement of this progress due to the fact that there are organizations that are exclusively run by men who work against FC in Norway. Hence, recruiting men in FC eradication programmes has a paramount importance for the successful eradication of FC in Somalia.

The present study has limitations. The circumcision status of female participants was determined through self-reported circumcision, which can be a subject to both over- and under-reporting. The study respondents were overrepresented by young people, and the sample was both urban, and to a degree, also educated. Thus, we know little about the attitude of old people towards the practice, which can mainly be attributed to the study’s season. It was conducted during the month of Ramadan, in which most elderly people stay away from big towns and prefer to fast in villages and nomadic hamlets. Secondly, the FC topic was a very sensitive issue in the study’s settings, which may then influence the participant’s response. However, females were interviewed by female assistants, while men were interviewed by other men, thus reducing the gender-related sensitivity of the topic. Furthermore, it was a cross-sectional study, thus limiting our ability to decide a cause and effect relationship. The study was limited in scope because it was conducted in the capital town, while the majority of people in Somaliland live in nomadic settings and villages.

## Conclusion

This study shows that people’s support towards the continuation of the practice is profoundly high in Somaliland. Among the factors associated with support for the continuation of FC is the belief that the practice is a religious requirement and that the *Sunna* form is not harmful. Accordingly, since religion was a significant factor behind the positive attitude towards the practice, religious interventions should be prioritized.

Men are more likely to have a positive attitude towards the practice than women; hence, awareness campaigns should equally target men, as it is widely known that without men’s involvement in eradication programmes, the efforts toward the abolishment of the practice may have little chance of success. Men’s involvement is critical in the efforts towards the abandonment of FC, although men may not be the target of the information, education and communication campaigns in Somaliland, nor have they played a key role in campaigns against FC. It is not an easy job for a Somali female campaigner to talk about the disadvantages of FC to their male counterparts; thus, in order to target men, the institutions that run FC programmes should also include men, who can be used to influence other men’s attitudes toward the practice.

Lastly, the study shows that the prevalence and support towards the practice among the people of Hargeisa is similar to the prevalence recorded in Somalia three decades ago. This demands an alternative approach other than continuing the status quo. To find an alternative approach that can lead to the total eradication of FC in Somalia, broad discussions should be organized among all stakeholders, such as international agencies, local communities that include religious leaders, politicians, civil society organizations, local women organizations, etc. An advocacy directed towards eradicating female circumcision is a cultural negotiation in which the method used is important [31]. The alternative approach towards the eradication of FC in Somalia should come from the local community, and its implementation should be their responsibility.

## Competing interest

Authors declare that they have no conflict of interest.

## Authors’ contributions

AG wrote the research protocol, initiated the field work, did the data analysis and drafted the manuscript. BB was involved in the fieldwork, data analysis and drafting of the manuscript. JS was involved in the write-up of the research protocol, the fieldwork, the data analysis and drafting of the manuscript. All authors read and approved the final manuscript.
